# A Primer of Psychology and Mental Disease

**Published:** 1899-12

**Authors:** 


**A Primer of Psychology and Mental Disease.** By C. B. Burr, M.D. Second Edition. Pp. ix., 116. Philadelphia: The F. A. Davis Company. 1898.

We seriously question whether it is requisite to give instruction in so peculiarly an abstract study as that of psychology to nurses and attendants, for whom this book has been written. So far, however, as this portion of the book is concerned, it matters perhaps but little whether the crude intelligence of the average nurse or attendant attacks it or not; on the whole, we should prefer a state of ignorance to that of mystification.

But when teaching on clinical insanity is attempted to this class of student, it is obvious that only well-considered and carefully-expressed statements should be made. As an instance of the failure to appreciate this, we may cite the assertion made at page 37, "In Mania there is no tendency to suicide." We can find nothing which serves to exclude puerperal mania, and, indeed, the only mention made of this condition is under the section of causation. Such an omission is serious. Again, under the head of what the author pleases to call "melancholia simple," acute and delusional forms are described, and the tendency to suicide is said to be strong. It is surely unwise not to qualify a statement which tends to oppress the nurse with needless responsibility in all degrees of melancholia alike. On the other hand we are informed that in melancholia with stupor there is not, as a rule, a tendency to suicide; this assertion betrays as much lack of caution as the previous one shows excess of it. The author speaks of dementia after mania and melancholia as a condition of temporary impairment: it is not wise to use this term loosely; dementia as properly understood implies an irrecoverable reduction in mind. Under the section, epileptic dementia, we meet with the truly surprising remark that just before, *during* (the italics are ours), or immediately following epileptic convulsions there are apt to be great mental confusion, irritability, and impulsiveness; this is as happily expressed as the statement in the next paragraph, that "epileptic patients are apt to be untidy, especially during convulsions"! Later on we learn that an objection to rest in bed is the danger of suicide.

It is not needful to quote further excerpts from this book; the author has not realised the great difficulties attending the production of a manual suitable for the nursing staff of an asylum, or grasped the necessity for sound and indisputable, even though brief, statements, and this want of careful purpose has gravely blemished a work which in many parts contains useful enough matter.

